# Translating orthopaedic basic science into clinical relevance

**DOI:** 10.1186/s40634-014-0005-x

**Published:** 2014-06-26

**Authors:** Henning Madry

**Affiliations:** Center of Experimental Orthopaedics, Saarland University, Homburg/Saar, D-66421 Germany

**Keywords:** Basic science, Orthopaedics, Translation, Clinics

## Abstract

In orthopaedic and trauma surgery, the rapid evolution of biomedical research has fundamentally changed the perception of the musculoskeletal system. Here, the rigor of basic science and the art of musculoskeletal surgery have come together to create a new discipline -experimental orthopaedics- that holds great promise for the causative cure of many orthopaedic conditions. The Journal of Experimental Orthopaedics intends to bridge the gap between orthopaedic basic science and clinical relevance, to allow for a fruitful clinical translation of excellent and important investigations in the field of the entire musculoskeletal system.

The essence of life science is to systematically describe the way organisms live. In the orthopaedic field, the rapid evolution of biomedical research during the past 30 years has changed fundamentally the way we think about the musculoskeletal system in health and disease. For example, while menisci were once thought to be functionless remnants of intra-articular leg muscles [1], it is now clear that these unique fibrocartilaginous structures have distinct functions, and multiple and serious efforts are devoted by orthopaedic sports medicine surgeons, biomaterial scientists, cell, molecular, and developmental biologists, biochemists, biotechnologists, and biomechanical engineers to preserve and to regenerate meniscal tissue [2–19] as a way to halt the initiation and progression of osteoarthritis [20,21]. Here, the art of musculoskeletal surgery and the rigor of basic science have come together to create a new discipline - experimental orthopaedics – that holds great promise for the causative cure of many musculoskeletal conditions that are not only disabling such as osteoarthritis, but are also life-threatening as the many bone and soft tissue tumours.

The founders of the *Journal of Experimental Orthopaedics* – the board of ESSKA, the European Society of Sports Traumatology, Knee Surgery and Arthroscopy – recognized the need to establish a scientific journal that would publish basic science reports of new and important investigations in the field of the entire musculoskeletal system with a view of translating them into clinical reality. ESSKA hosts bi-annual congresses, with an ever-growing number of participants and presentations of high scientific quality. Of note, ESSKA has a long tradition of supporting basic science, highlighted in the prestigious Theo van Rens Award for the best scientific presentation, named after Professor Theo J. G. van Rens (1931–1986), one of the founding members of ESSKA [22]. The *Journal of Experimental Orthopaedics* intends to bridge the gap between orthopaedic basic science and clinics. Its goal is to publish papers related to the entire field of experimental orthopaedics, including physiological, pathological, and therapeutic aspects of cartilage, bone, tendons, ligaments, and other musculoskeletal tissues. Scientific excellence is the main criterion for publication. Another basic principle is to provide a peer review process that is both decisive and fair to authors, with a focus of a fast turnaround time. As we believe that the results of scientific research should be freely available to everyone to read and use, we have chosen an open access model, which is, among others, supported by such highly prestigious organizations such as the European Commission, the Howard Hughes Medical Institute, the Max Planck Society, the National Institutes of Health and the Wellcome Trust. The international *Journal of Experimental Orthopaedics* will thus enable clinicians and scientists to better work together in order to take a leading role in the publication of research in a way that is without restricted access to these results.

At the *Journal of Experimental Orthopaedics*, our goal is to put high quality basic science first, as the editorial decision making process is not driven by profits or scientific fashions but by pure academic excellence. We envision papers that are exciting, clearly written, and based on original data with a clinical background. In addition to research articles, the journal provides a forum to publish both comprehensive and focused reviews.

The *Journal of Experimental Orthopaedics* is thought to complement the esteemed *Knee Surgery, Sports Traumatology, Arthroscopy* journal, which focuses on clinical investigations. When *Knee Surgery, Sports Traumatology, Arthroscopy* was launched in 1993, Ejnar Eriksson, its founding editor, asked the question “Why another new scientific journal when there are already so many” [23]? Now, 21 years later, KSSTA ranks among the top 33% of journals in both Orthopaedics and Sports Sciences. I have no doubt that the *Journal of Experimental Orthopaedics* with its excellent Editorial Board, together with its distinguished Board of Trustees, and its committed editorial team, will similarly flourish in the wake of such visionaries like Eriksson, van Rens, and many others, and invite you to select the *Journal of Experimental Orthopaedics* as your first choice to publish your best orthopaedic basic research.
